# Machine Learning-Driven Refinement of Reactive Force Fields via Hierarchical “Center-Environment” Features for Energetic Molecular Crystals

**DOI:** 10.3390/molecules31162814

**Published:** 2026-08-12

**Authors:** Qi He, Pengju Wang, Xudong He, Jincang Zhang, Yi Liu

**Affiliations:** 1 Materials Genome Institute & State Key Laboratory of Materials for Advanced Nuclear Energy, Shanghai University, Shanghai 200444, China; heqi@shu.edu.cn (Q.H.); hxd_yi@shu.edu.cn (X.H.); jczhang@shu.edu.cn (J.Z.); 2 Zhejiang Laboratory, Hangzhou 311100, China

**Keywords:** machine learning, Δ-learning, transfer learning, Hierarchical “Center-Environment” features, ReaxFF force field refinement, energetic molecular crystals

## Abstract

Accurate energy prediction in organic molecular crystals, such as cyclotrimethylenetrinitramine (RDX) and cyclotetramethylenetetranitramine (HMX), is hindered by the scarcity of descriptors capable of encoding their hierarchical architecture. Furthermore, while reactive force field (ReaxFF) offers a pathway to simulating chemical reactions, its accuracy for polymorph stability remains suboptimal, and machine learning (ML) driven refinement of ReaxFF for molecular systems remains unexplored. Herein, we report the first construction of a Hierarchical “Center-Environment” (HCE) feature framework specifically tailored for molecular crystals. The HCE framework hierarchically decomposes structural complexity into intramolecular (ring vs. nitro groups) and intermolecular (central molecule vs. coordination shell) attributes, integrating physics-based priors with distance-weighted attention. Using this compact descriptor set, we present the first attempt to rectify ReaxFF predictions via ML, benchmarking against 5930 RDX and 3335 HMX DFT-calculated energies. Our models achieve a significant reduction in mean absolute error: from ~116.7 to 42.2 meV/atom for RDX (kernel ridge regression, KRR) and from 112.3 to 53.7 meV/atom for HMX (support vector regression, SVR). Crucially, HCE features enable robust cross-molecule transferability; a neural network pre-trained on RDX yields a validation error of 52.9 meV/atom on HMX, surpassing models trained exclusively on HMX data. This work not only establishes a pioneering paradigm for interpretable ML-driven force field refinement but also provides the first feature engineering solution incorporating chemical, physical, and structural information specifically designed for the machine learning of energetic molecular crystals.

## 1. Introduction

Energetic materials are extensively used in civilian, aerospace, and military applications, and are mainly composed of organic molecular crystals containing CHNO elements [1,2]. Nitramine energetic crystals such as cyclotrimethylenetrinitramine (RDX) [3,4] and cyclotetramethylenetetranitramine (HMX) [3,5,6] exhibit good thermal stability, high energy, and high detonation velocities, making them the primary energy sources in military explosives and propellants. However, like many organic solids, energetic crystals frequently exhibit polymorphism due to their complex molecular conformations and intermolecular packing arrangements [6,7,8]. For instance, RDX [8,9,10,11] and HMX [8,12,13,14] are known to possess five and four polymorphs, respectively. These structural variations significantly influence key properties, including density, sensitivity, and mechanical properties [8,15]. Consequently, it is essential to elucidate the relationship between crystal structures and their energetic stability and properties.

Polymorphic transitions in energetic crystals can occur throughout their entire lifecycle, encompassing manufacturing, storage, and application. For example, the α → β transition of RDX can be consistently obtained via drop-cast crystallization from various organic solvents, while β-RDX can remain stable for over a year [16]. HMX undergoes a β → δ transition induced by thermal stress, which significantly affects the thermal stability and impact safety of HMX-based polymer-bonded explosives [17]. Therefore, inhibiting polymorph transitions remains a significant challenge and a major research focus [18,19,20,21,22]. Although phase diagrams have been established experimentally [9], and macroscopic phenomena associated with phase transitions—such as thermally induced cracking in HMX [23] and pressure-induced structural transformation in β-RDX [24]—have been extensively observed, the underlying microscopic mechanisms governing these transformations remain to be fully elucidated.

Computational simulations have thus become the primary approach for exploring polymorphic transformations in energetic materials. Among these, first-principles calculations are favored for their high accuracy. In recent years, density functional theory (DFT) combined with dispersion corrections has proven effective in simulating intermolecular interactions and has become the mainstream method. For instance, DFT calculations have been successfully employed to investigate the high-pressure phase transition and compressibility of RDX [25], as well as the pressure- and temperature-dependent structural, vibrational, and thermodynamic properties of NTO crystals [26,27]. However, the inherent complexity arising from the large-scale, multi-atomic nature of energetic molecular crystals poses significant challenges for DFT calculations, hindering theoretical progress. Hence, methodological breakthroughs are urgently needed [28].

To address these challenges, some researchers have employed empirical potentials or classical force fields [29,30]. For α-RDX, Sorescu et al. developed an intermolecular potential that yielded lattice parameters in excellent agreement with experiments and formation energies in reasonable agreement [29]. Subsequently, they applied this potential function to study over thirty other molecular crystals containing nitrate groups. By incorporating atomic charge corrections that account for electronic correlation effects, most of the computed crystal structures agreed with experimental results. However, discrepancies were still observed in the calculated formation energies compared with experimental values [30]. Alongside such customized potentials, several general-purpose force fields—including Lennard-Jones [31], CHARMM [32,33], and COMPASS [34]—have also been widely applied to energetic material systems. Beyond static calculations, molecular dynamics (MD) simulations are required to understand crystal behavior at finite temperatures and pressures, with the ReaxFF reactive force field [35] being widely used to study decomposition processes under shock conditions [36,37,38]. Despite its wide applicability, the accuracy of ReaxFF remains a subject of ongoing improvement. For instance, the ReaxFF-lg [39] force field was developed to better describe London dispersion interactions, which are critical for the accurate prediction of equations of state for energetic materials. More recently, the ReaxFF_CHO_-S22 [40] model achieved enhanced accuracy for C/H/O systems through refined parameterization. However, even with these advancements, the quantitative accuracy of ReaxFF in predicting the energies of complex molecular crystals such as RDX and HMX remains limited compared with first-principles methods.

In recent years, machine learning (ML) has emerged as a powerful tool in computational materials science and has been widely applied in this field [41,42,43,44,45,46,47,48]. Omee et al. combined a multi-objective genetic algorithm with a neural network interatomic potential for efficient crystal structure prediction, demonstrating superior performance over traditional methods across diverse benchmark materials [49]. In addition, Li et al. systematically reviewed recent advances in machine learning-assisted crystal structure prediction, covering graph neural networks, machine learning force fields, and generative models [50]. Beyond conventional property prediction, transfer learning has recently been explored to extend ML capabilities across different material systems. For instance, Fang et al. fine-tuned a pretrained MACE foundation model for Mo-based dilute alloys containing 20 substitutional elements, achieving energy prediction errors as low as 2.27 meV/atom—a 7- to 20-fold improvement over models trained from scratch or zero-shot foundation models [51]. Their work demonstrates the efficacy of transfer learning from globally ordered homogeneous systems to locally ordered heterogeneous multicomponent alloy environments, although direct extrapolation to entirely unknown elements remains challenging [51].

Organic molecular crystals, with their complex conformations and packing, pose particular challenges for feature engineering in machine learning [7,8]. Clements et al. used the smooth overlap of atomic positions (SOAP) [52,53] and atom-centered symmetry functions [42,54] to characterize molecular crystals [55]. Gaussian process regression (GPR) [44] combined with first-principles data has achieved high-precision energy predictions [56,57,58]. Wengert et al. extended SOAP + GPR to correct energies and forces in molecular crystals, outperforming DFTB [59,60]. Kapil and Engel combined electronic structure with neural networks to compute free energies of organic crystals [61]. However, the current feature engineering representations focus either on nonperiodic molecules or periodic crystals, but not on both features of molecular crystals simultaneously. These approaches rely on high-dimensional descriptors and large datasets, incurring high computational and generalization costs, and most of them have yet to be validated for energetic molecular crystals. Nevertheless, recent studies have begun to explore ML-driven approaches for nitramine-based energetic materials. For instance, Ye et al. combined evolutionary algorithms with machine learning potentials to search for new cocrystal structures of CL-20 and HMX, demonstrating the feasibility of data-driven methods for this class of materials [41].

Despite the notable progress made by the above studies, two critical research gaps remain in current machine learning modeling of energetic molecular crystals. First, most feature description methods are designed either for isolated molecules or for periodic atomistic crystals in isolation; there is a lack of a unified description framework that can simultaneously capture intramolecular conformational flexibility and intermolecular packing environments. This deficiency prevents models from comprehensively reflecting the hierarchical structure–property relationships inherent to energetic crystals. Second, for error correction of empirical force fields such as ReaxFF, existing Δ-learning strategies predominantly rely on high-dimensional global descriptors. In energetic materials, which exhibit strong anisotropy and significant non-bonded interactions, these approaches lack physically meaningful and data-efficient local environmental features, leading to limited generalizability and interpretability of the correction models. To fill these gaps, the following specific scientific questions urgently need to be addressed: (1) How can we construct a hierarchical feature descriptor that encodes both intramolecular chemical bonding and conformational changes, as well as intermolecular packing and van der Waals interactions? (2) How can we use such a descriptor to accurately predict the systematic errors of ReaxFF relative to DFT with a small training dataset, thereby achieving first-principles accuracy at a fraction of the computational cost?

In contrast to these data-demanding approaches, our group has developed a “Center-Environment” (CE) feature model [62] that encodes local atomic environments into physically meaningful descriptors via predefined attention weighting, reducing data requirements while enhancing interpretability. The CE model constructs feature vectors by treating a critical atom as the “center” and its surrounding coordination shell as the “environment,” with features weighted by the reciprocal of the distance between the center and environment atoms. This framework has been successfully validated across diverse inorganic systems—including spinel/perovskite oxides for electrocatalysis [63,64,65,66], Co/Ni-based superalloys for superalloys applications [67,68,69,70], Nb-based alloys for ultrahigh-temperature structural materials [71,72,73,74,75], and nanocarbon nanomaterials on metal surfaces [76]—demonstrating its generalizability and data efficiency in small-data scenarios. The methodological principles and broad applicability of the CE framework have been systematically reviewed in a recent publication [62]. However, organic molecular crystals present new challenges that cannot be captured by atom-centered features alone: the hierarchical nature of molecular structure—encompassing both intramolecular conformation and intermolecular packing—requires a feature model that operates at the molecular rather than the atomic scale. This motivated us to extend the CE framework from inorganic atomistic crystals to molecular crystals, leading to the “Hierarchical Center-Environment” (HCE) feature model proposed in this work.

In light of the above considerations, in this paper, we perform first-principles calculations on the obtained RDX and HMX crystals to acquire their energy information as training targets for ML. Given the inherent accuracy limitations of empirical force fields, instead of pursuing further force field reparameterization, we adopt a complementary Δ-learning strategy: using machine learning to model and correct the systematic errors of ReaxFF relative to DFT, thereby achieving DFT-level accuracy at a fraction of the computational cost. Specifically, based on the CE framework [63,64,67,76], we decompose the feature model into intramolecular and intermolecular features. Leveraging physical knowledge and attention mechanisms, we construct HCE descriptors and train multiple ML models to predict the errors of ReaxFF compared with DFT. Then the ML-predicted errors are used as a correction to the original ReaxFF for improved accuracy.

The significance of this work extends beyond providing a high-accuracy, low-cost energy prediction scheme for RDX and HMX. More importantly, we establish a generic hierarchical feature modeling framework that can be extended to other organic molecular crystal systems. By embedding the physical priors of intramolecular and intermolecular interactions into the data-driven model, we effectively enhance the interpretability and small-sample learning capability of machine learning force fields, offering a new route to resolve the long-standing dilemma between accuracy and efficiency in simulating polymorphic transitions of energetic materials. Furthermore, the proposed HCE descriptors and Δ-learning correction strategy are expected to accelerate the study of key issues such as high-pressure phase transitions, thermodynamic stability, and sensitivity mechanisms of energetic crystals, and to lay a methodological foundation for future ML-driven intelligent screening and design of energetic molecular crystals. This work establishes a feature description method for energetic materials and a data-driven accuracy enhancement method for ReaxFF, offering new pathways for ML force field development, polymorph simulation, and intelligent design of organic molecular crystals.

## 2. Methods

### 2.1. Dataset Collection

First, a combination of Monte Carlo and simulated annealing algorithms [77,78] was employed alongside the MORS6-SCFF [79] force field to search for crystal structures of RDX and HMX across 48 space groups, ranging from space group 14(P21/c) to 61(Pbca), a range that covers the most representative space group types in organic crystals, aiming to explore potential new stable crystal phases beyond the known ones and our previous work [28]. Second, the ReaxFF-lg [39] force field was used for geometry optimization, energy evaluation and electronic population via the Large-scale Atomic/Molecular Massively Parallel Simulator (LAMMPS, version 15 June 2023) package [80]. This procedure yielded 289 RDX and 165 HMX crystal structures.

To enrich the dataset for ML training, we performed additional geometric deformations on the 454 optimized structures, including compression, shear, lattice parameter variation, and dihedral angle rotation, followed by re-optimization and subsequent DFT calculations using the Vienna Ab initio Simulation Package (VASP, version 5.4.4) [81]. The crystal structures in the dataset were filtered using the following criteria to eliminate unreasonable configurations: (1) structures with total energies more than 700 eV (for RDX) or 1000 eV (for HMX) above the lowest-energy structure; or (2) structures with any C–N, N–O, or C–H bond lengths deviating by more than 20% from experimental values. This screening ensured that only physically and chemically reasonable structures were retained for subsequent calculations and model training. After screening out unreasonable structures, the final dataset contained 5930 RDX and 3335 HMX crystal structures.

All DFT calculations were performed using the generalized gradient approximation with the Perdew–Burke–Ernzerhof (PBE) functional [82]. The projector augmented-wave (PAW) [83] was employed with a kinetic energy cutoff of 1000 eV [84,85], and a Monkhorst–Pack k-point mesh of 2 × 2 × 2 was used to compute the energies and atomic forces of the aforementioned 9265 structures. All calculations were carried out using the VASP, version 5.4.4. The electronic self-consistent field (SCF) convergence criterion was set to 1 × 10^−4^ eV, and ionic relaxations were performed until the maximum force on each atom fell below 0.01 eV/Å. Grimme’s DFT-D3 dispersion correction [86] was applied to account for long-range van der Waals interactions. All energy values presented herein are total energies per primitive cell (in eV), whereas the RMSE values are provided in meV/atom, corresponding to the total energy error divided by the number of atoms.

### 2.2. HCE Framework

**Figure 1:**
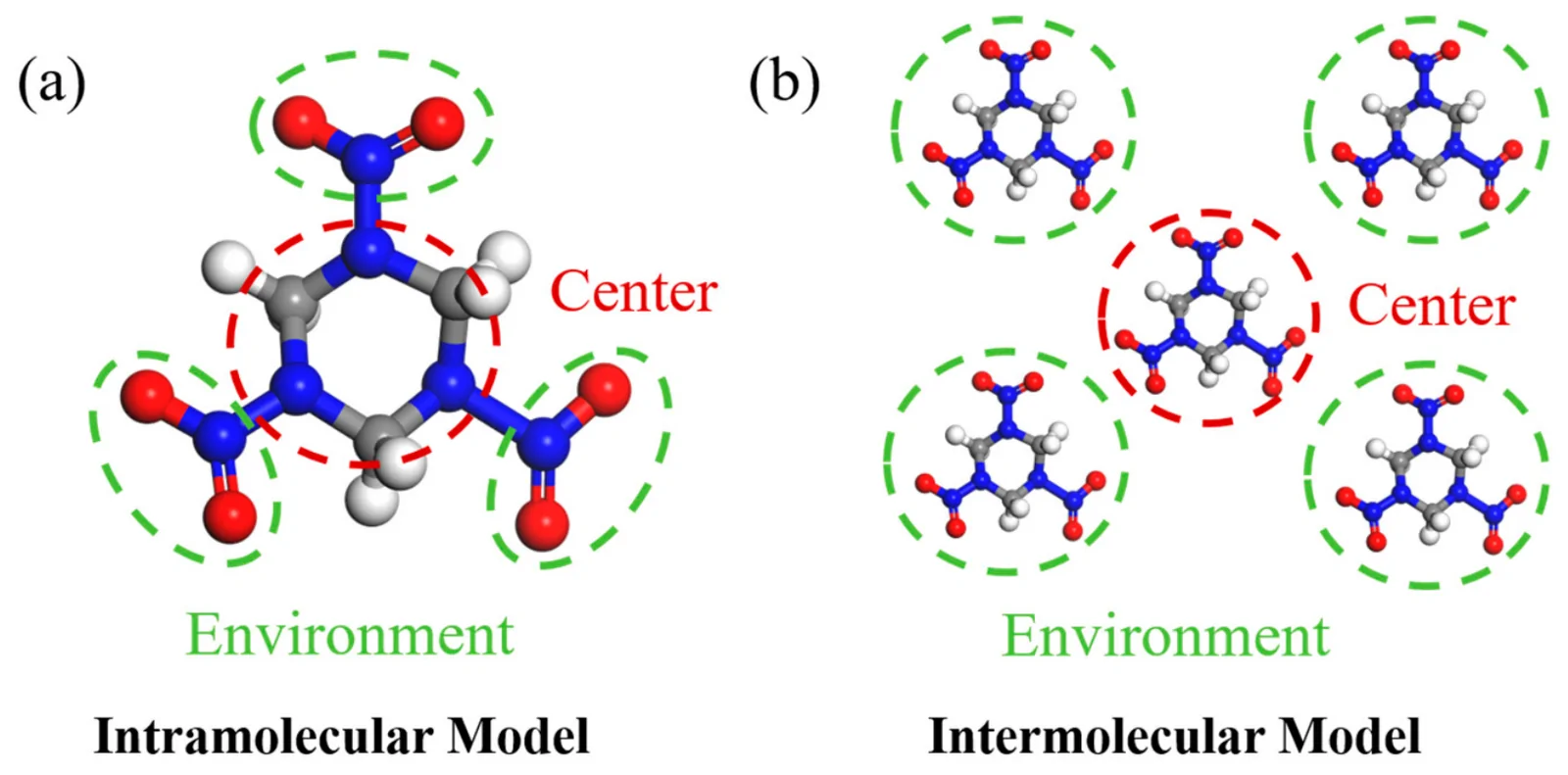
Schematic diagrams of (**a**) the intramolecular model and (**b**) the intermolecular model of the hierarchical “Center-Environment” feature model. The “center” in (**a**) is the central C–N ring, and in (**b**) it is the central molecule, with the neighboring molecules serving as the environmental components.

As shown in Figure 1, the HCE framework is disaggregated into a hierarchical architecture of intramolecular and intermolecular models. Within this framework, we encode each model’s structure distinctly.

In the intramolecular feature model, the C–N rings of RDX/HMX molecules serve as the center. Thus, the structural features (such as C–N bond length, C–H bond length and N–C–N bond angle), and calculated features (such as populations of C/H/N atoms of the rings), are chosen as potential descriptors for this central group (Figure S1 of the Supplementary Materials).

The nitro groups (-NO_2_) are treated as the intramolecular environmental components (Figure 1a). A predefined attention mechanism is incorporated where the reciprocal of the distance between the nitro group and the central C–N ring (i.e., the N–N bond length) is utilized as a weighting factor for features, alongside an unweighted counterpart for comparative analysis. For the environmental groups, fundamental elemental features are complemented by structural characteristics such as N–O bond length, O–N–O bond angle, and the dihedral angle between the nitro group and the best-fit plane of the C–N ring. Additionally, the information pertaining to atomic and group charges and polarization effects, which is also included as descriptors for the environmental features (Figure S2 of the Supplementary Materials).

For the intermolecular feature model, each molecule in the crystal is taken as the center, and features such as the area of polygons formed by the N atoms in the nitro groups and dipole moments calculated by ReaxFF serve as descriptors (Figure S3 of the Supplementary Materials).

The molecules within the first nearest-neighbor shell surrounding the central molecule are identified as environmental molecules (Figure S4). In order to locate these first nearest-neighbor shell molecules, a molecular environment classification algorithm based on the Atomic Environment Type (AET) method was developed [73,74]. The AET-based environmental features, originally used by our group for crystalline materials and extended here to molecular crystals, define a physically closed environmental shell through geometric-topological rules—specifically, the maximum distance gap and convex volume criteria—rather than arbitrary distance cutoffs. This method ensures that the selected environmental molecules constitute a complete, non-redundant set determined by the geometry of the molecular crystal lattice [73,74]. In this work, we extend the concept of Atomic Environment Type (AET) to Molecular Environment Type (MET) to describe molecular crystals. Beyond the characteristics of the central molecule, additional features are selected, including the angle between the normal vectors of the C–N rings of the environmental and central molecules, as well as the dipole moment angles. These features are weighted by the reciprocal of the distance between the geometric centers of the environmental and central molecules and a constant unit value, respectively (as depicted in Figure S4 of the Supplementary Materials).

After implementing the aforementioned steps, a total of 61 features have been extracted. These features, integrating both intramolecular and intermolecular models, form the HCE feature model. This one-dimensional feature vector ***d*** for any given structure can be represented as [73,74]:

(1) $$d=[d_{1},d_{2},d_{3}\ldots d_{m}]$$

Among them, m represents the number of selected basic properties. If *d_i_* is the central feature:

(2) $$d_{i}=p_{i}$$

If *d_i_* is an environmental feature:

(3) $$d_{i}=\sum_{j=1}^{n}\omega_{j}p_{j,i}$$

For intramolecular models, *n* represents the number of nitro groups in the molecule; for intermolecular models, *n* represents the number of first-nearest-neighbor molecules corresponding to the central molecule, and *ω*_j_ is the weight of the *j*th nitro group/molecule. In this work, we employ two different weighting schemes. The first is *w*_i_ = 1/*n*, and the second can be calculated using the following formula:

(4) $$\omega_{i}=\frac{1/r_{i}}{\sum_{j=1}^{n}1/r_{j}}$$

For intramolecular models, *r_j_* represents the distance between the *j*th nitro group and the C–N ring, i.e., the N–N bond length. For intermolecular models, *r*_j_ represents the geometric center distance between the *j*th environmental molecule and the central molecule. The feature vector set ***V***, composed of the material data set, can be expressed as:

(5) $$V=[d_{1},d_{2},d_{3}\ldots d_{N}]$$

where *N* represents the number of samples in the data set. Subsequently, all elements in the feature set ***V*** are normalized to obtain a dimensionless feature set.

The choice between uniform weighting (*w*_i_ = 1/*n*) and inverse-distance weighting (Equation (4)) reflects two different physical assumptions about the dominant interaction in the crystal. The uniform weighting treats all environmental groups and molecules equally and may be more appropriate when long-range collective effects (such as lattice strain or polarization fields) dominate. In contrast, the inverse-distance weighting, motivated by the inverse-distance decay of electrostatic interactions, emphasizes the contribution of near-neighbor nitro groups and molecules. Both weighting schemes were evaluated across all ML algorithms; the optimal choice varied depending on the ML method and the target material system (see Section 3.3).

### 2.3. Machine Learning

Four methods, including linear regression (LR), random forest (RF), support vector regression (SVR) and extreme gradient boosting (XGBoost) were employed to rank the features. Features that appeared in the top 30 in importance by at least three of the four methods were combined into a new feature set.

SVR, RF, kernel ridge regression (KRR), and artificial neural networks (ANN) were employed to train the RDX and HMX crystal data sets. For SVR, RF and KRR, grid search methods were used to optimize their hyperparameters. For ANN, Bayesian optimization was employed to select the hyperparameters. The hyperparameters are listed for the ML models of RDX crystals in Table S1 of the Supplementary Materials and those of HMX crystals in Table S2 of the Supplementary Materials.

For each ML method, ten independent model trainings were conducted. In each training, 20% of the dataset was selected randomly as an independent test set, while the remaining 80% was used as a training/validation set with five-fold cross-validation. The average performance across the validation and test sets from the ten independent trainings is used as the basis for evaluating the model’s effectiveness.

Furthermore, to investigate the effectiveness of transfer learning between RDX and HMX data sets, the same features were selected for both RDX and HMX data sets. A new ANN model trained on the RDX data set is then transferred to the HMX data set, with all node weights adjusted accordingly, to evaluate the effectiveness of this transfer learning approach.

## 3. Results and Discussion

**Figure 2:**
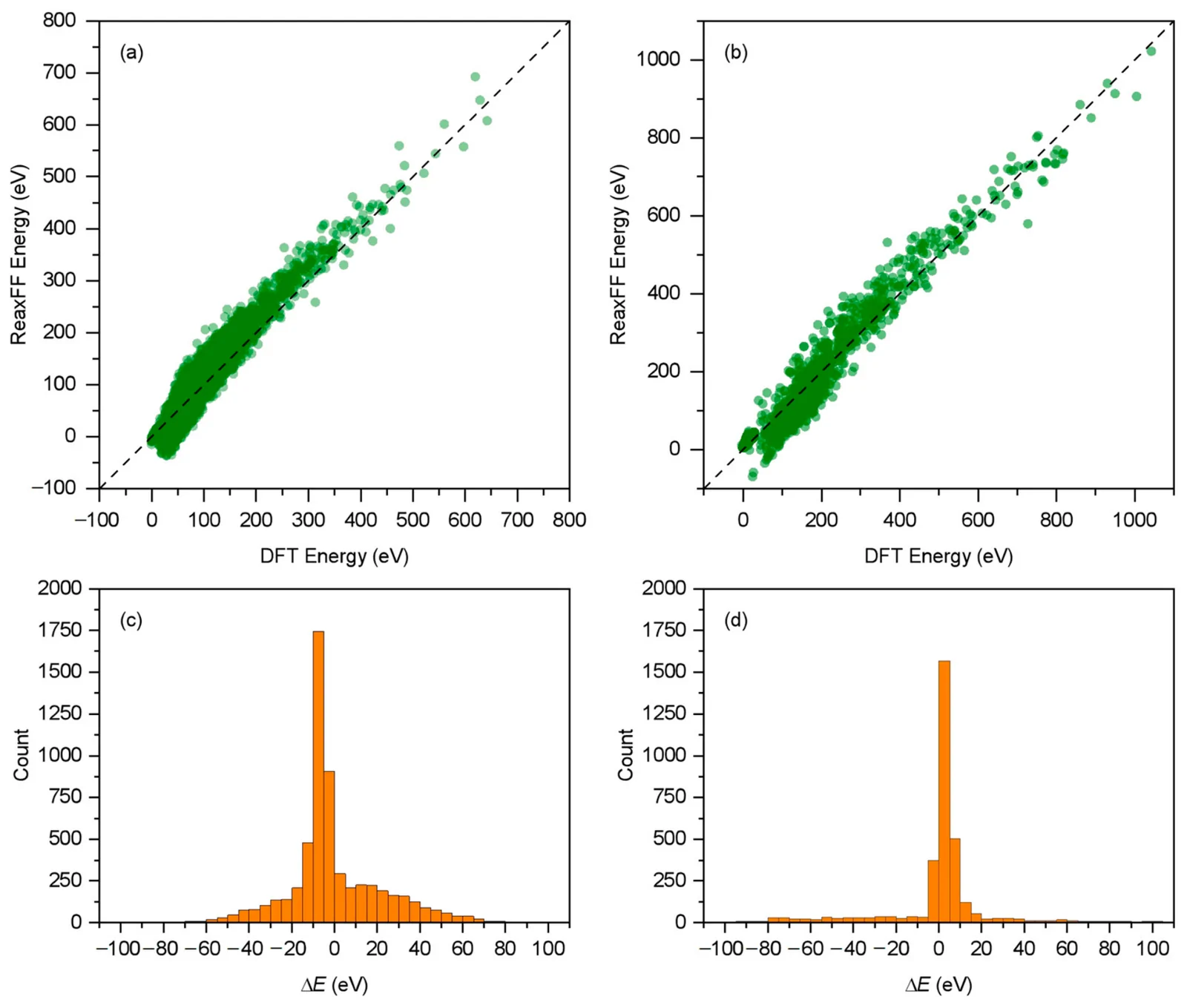
Correlation of ReaxFF energies with the corresponding DFT energies of (**a**) RDX and (**b**) HMX crystals; error distribution of (**c**) RDX and (**d**) HMX crystals.

In this paper, a ML model based on the HCE feature framework combined with ReaxFF is employed to simulate the energies of energetic organic molecular crystals as computed using DFT. The energies of RDX and HMX crystals calculated using ReaxFF and DFT are shown in Figure 2a and Figure 2b, respectively. It can be seen that the error for RDX crystals is approximately 116.7 meV/atom, while the error for HMX crystals is lower, at 112.3 meV/atom. However, as shown in Figure 2c,d, the error distribution for RDX crystals is closer to a normal distribution compared with HMX crystals, which indicates it is more suitable for ML training. Additionally, the data points are unevenly distributed on either side of the dashed line, indicating a systematic nonlinear deviation between the ReaxFF calculations and DFT results. This suggests that ReaxFF is insufficient for rapidly obtaining accurate energy information for RDX and HMX crystals. Therefore, there is a need for a method that can accurately determine material energies while saving the substantial time required for DFT calculations.

### 3.1. Feature Analysis

**Figure 3:**
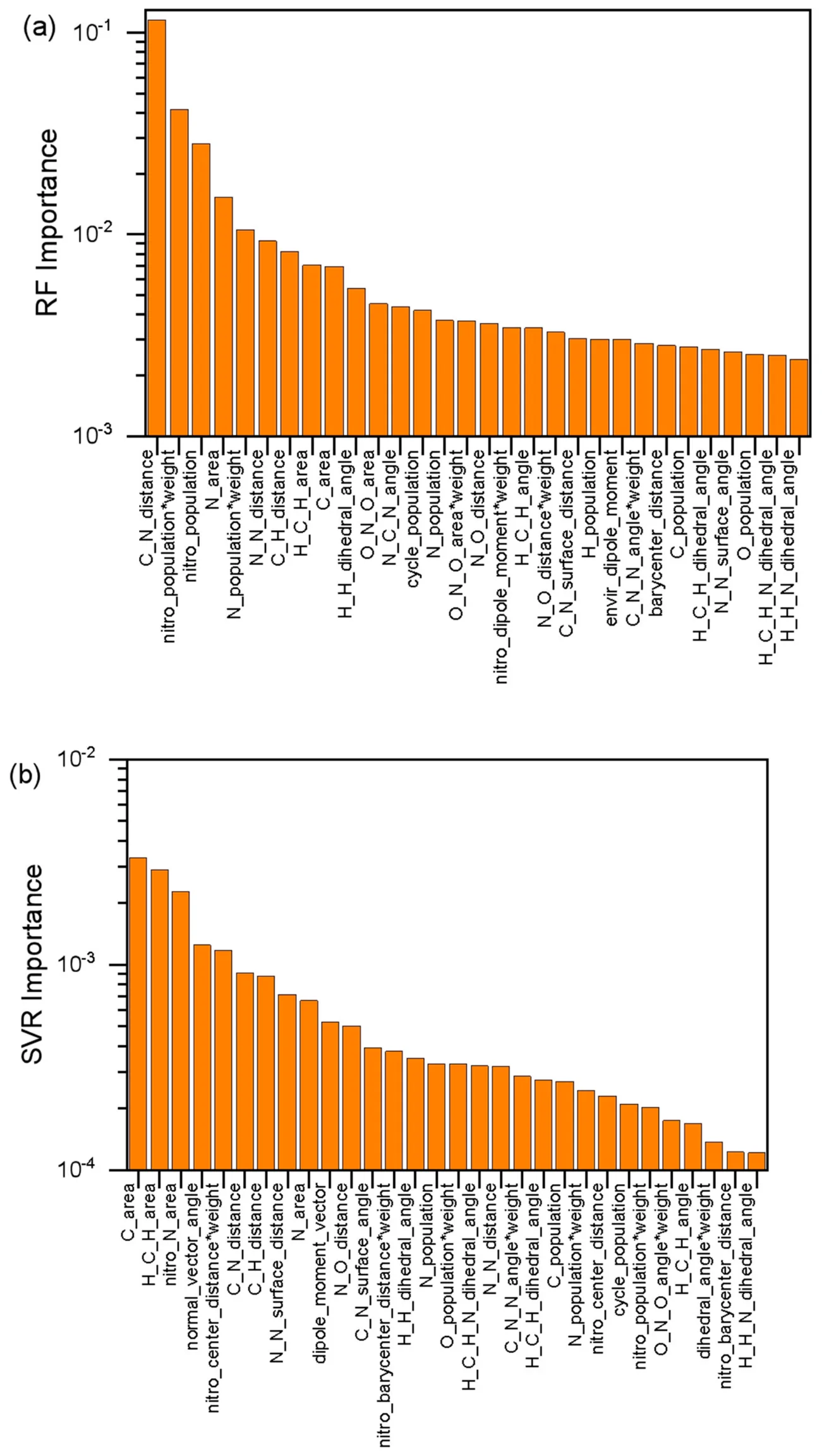

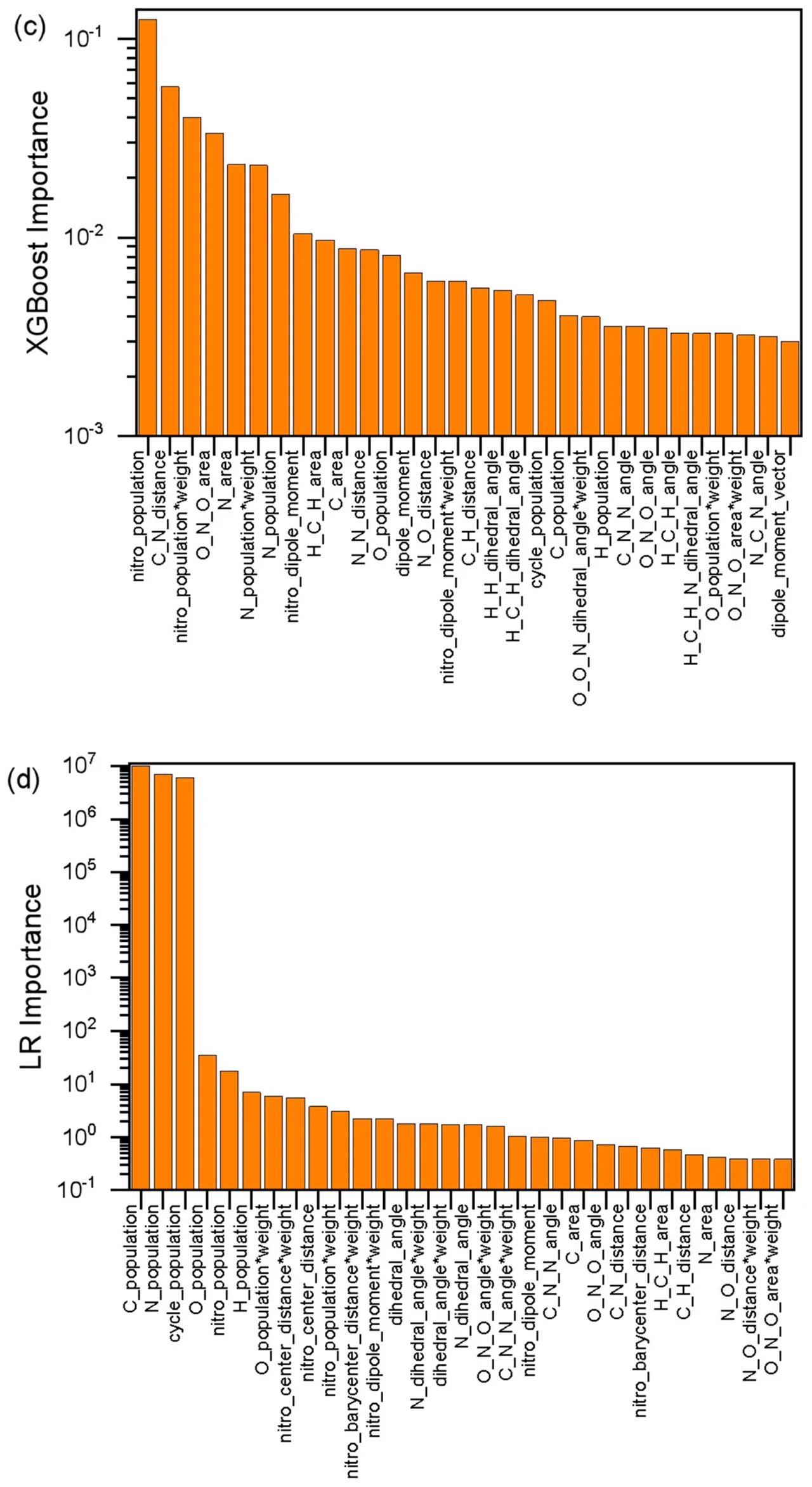
Features of RDX crystals with importance ranked in the top 30 by (**a**) RF, (**b**) SVR, (**c**) XGBoost and (**d**) LR methods, * weight represents the weight by reciprocal of distances.

As shown in Figure 3, for RDX crystals, 21 features such as C_area and C_H_distance are ranked among the top 30 in at least three of the four methods (RF, SVR, XGBoost, and LR). These features are selected as input features for machine learning training in this paper. Notably, eight features, including C_N_distance and C_population, are consistently ranked in the top 30 across all four ranking methods, indicating their more significant impact on structural energy.

**Figure 4:**
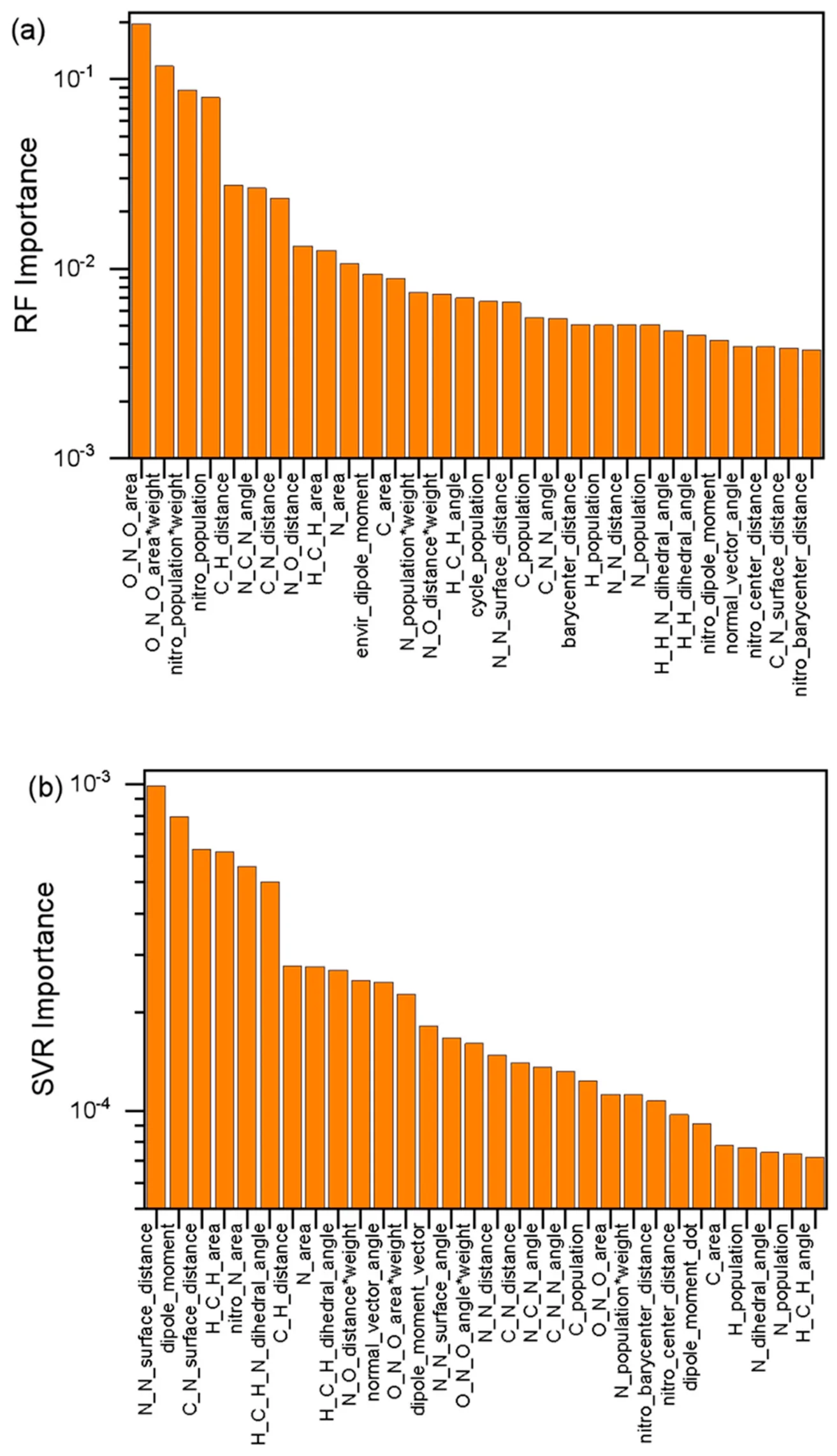

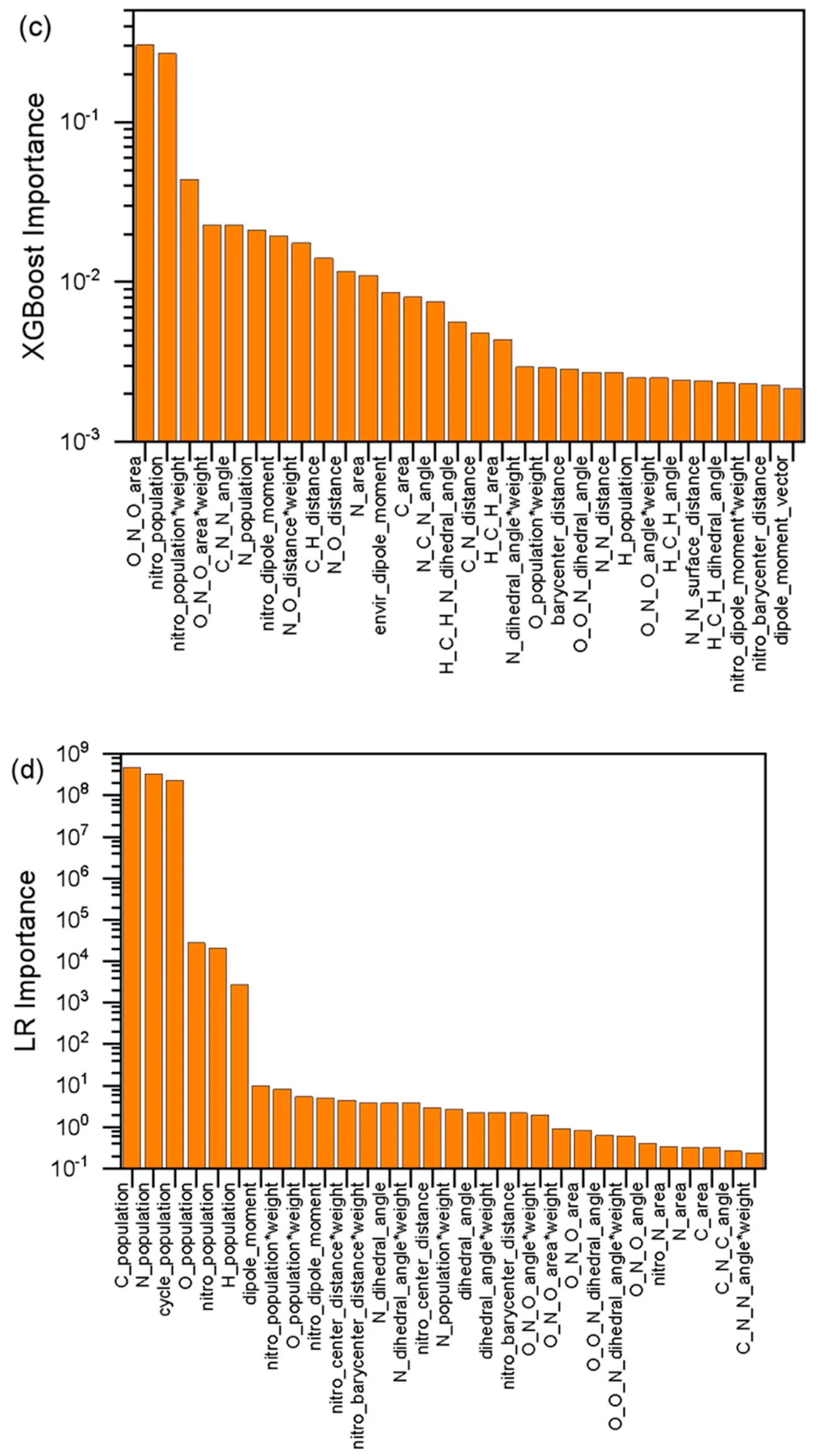
Features of HMX crystals with importance ranked in the top 30 by (**a**) RF, (**b**) SVR, (**c**) XGBoost and (**d**) LR methods, * weight represents the weight by reciprocal of distances.

Similarly, for HMX crystals as depicted in Figure 4, 22 features, such as C_area and C_H_distance, are ranked in the top 30 in at least three of the RF, SVR, XGBoost, and LR ranking methods. These features are chosen as input features for ML training. Among them, seven features, such as N_area and H_population, are consistently ranked in the top 30 across all four ranking methods, suggesting their significant influence on the structural energy of HMX.

### 3.2. ML Training

**Figure 5:**
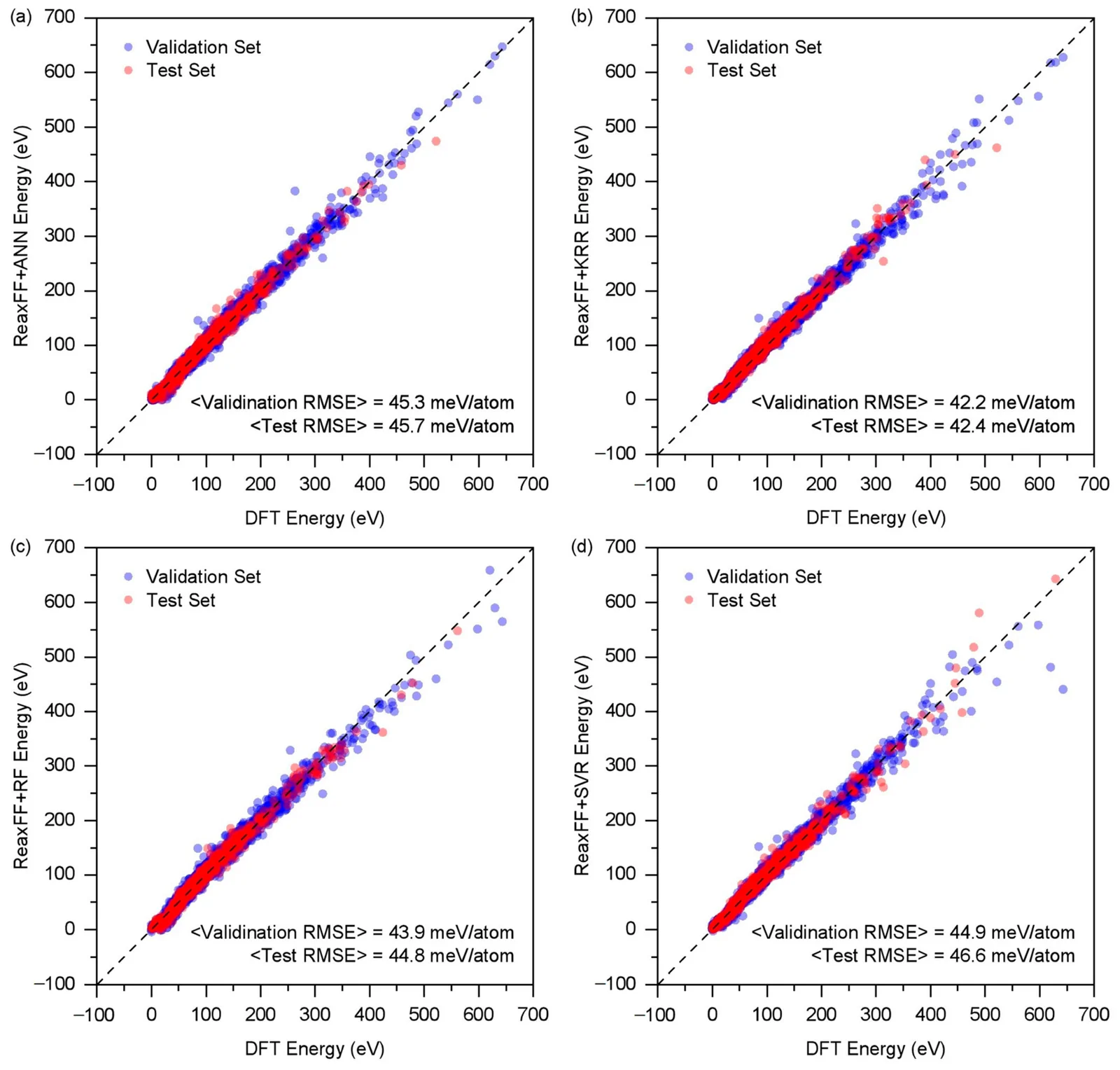
Correlation of ReaxFF + ML energies with the corresponding DFT energies for all structures in the validation (blue dots) and test (red dots) sets of RDX crystals by (**a**) ANN, (**b**) KRR, (**c**) RF and (**d**) SVR.

**Figure 6:**
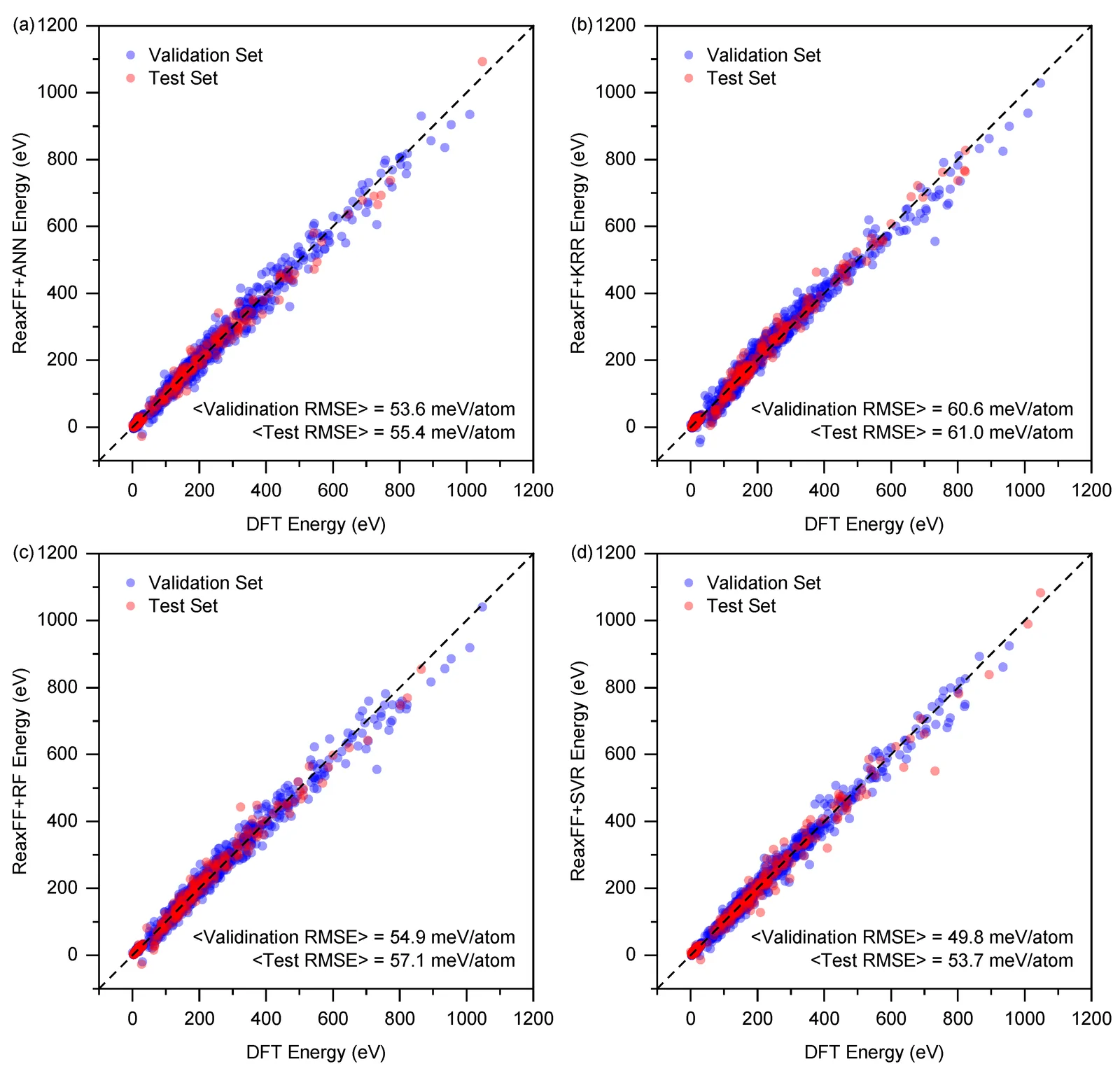
Correlation of ReaxFF + ML energies with the corresponding DFT energies for all structures in the validation (blue dots) and test (red dots) sets of HMX crystals by (**a**) ANN, (**b**) KRR, (**c**) RF and (**d**) SVR.

Subsequently, we employed four machine learning methods, namely ANN, KRR, RF, and SVR, to train the ML models of RDX and HMX. The ANN training, performed on a single NVIDIA RTX 4090 GPU, took approximately 1 h. By contrast, all other ML methods, including SVR, RF, and KRR, were executed on 16 CPU cores and each completed within a few minutes. The results of these training sessions are presented in Figure 5 and Figure 6 and Tables S3 and S4 of the Supplementary Materials, and the complete regression equations, Pearson correlation coefficients (R), and RMSE for all models on both validation and test sets are summarized in Table S7 of the Supplementary Materials.

Figure 5 compares the energies calculated by ReaxFF in conjunction with the models trained using ANN, KRR, RF, and SVR for RDX crystals against DFT calculations. It can be observed that the models trained by the four methods show similar performance, with closely matched errors between the validation and test sets, indicating no overfitting and the potential for generalization to a wider range of RDX crystal systems. Notably, the KRR model exhibits the smallest error, with average errors of 42.2 meV/atom and 42.4 meV/atom for the validation and test sets, respectively, which are the lowest among the four methods. After ML training, the corrected energies show an error that is approximately one-third of that obtained using the ReaxFF alone when compared with DFT results. This demonstrates that ML training based on the HCE model can effectively correct the errors between DFT calculations and force field predictions, thereby enhancing the accuracy of the force field.

Figure 6 presents a comparison of the energies calculated by the models trained using ReaxFF in conjunction with ANN, KRR, RF, and SVR for HMX crystals against DFT calculations for HMX crystals. It is evident that, compared with RDX crystals, the models trained for HMX crystals exhibit larger errors, and there are differences in the performance of the models trained using the four methods. Among them, the SVR model demonstrates the smallest error, with average errors of 49.8 meV/atom and 53.7 meV/atom for the validation and test sets, respectively, which are the lowest in the four methods. In contrast, the KRR method, which performed best for RDX crystal training, shows the worst results for HMX crystal training, with errors exceeding 60 meV/atom for both the validation and test sets. Compared with the error of 112.3 meV/atom obtained by ReaxFF, the error after ML training is approximately half.

### 3.3. Transfer Learning

Due to the poorer performance of the models trained on HMX crystals compared with those trained on RDX crystals, we transferred the ANN model trained on RDX crystals to HMX crystals for fine-tuning of parameters. However, because the different feature sets were selected for RDX and HMX crystals, the ANN model trained on the RDX crystal dataset cannot be directly applied to HMX crystals. To address this issue, we separately analyzed the features selected for RDX and HMX crystals in the previous section and merged the feature sets from both datasets, resulting in a total of 34 features. These features were then used comprehensively in the model training to ensure consistency for both crystal types, enabling successful transfer learning.

**Figure 7:**
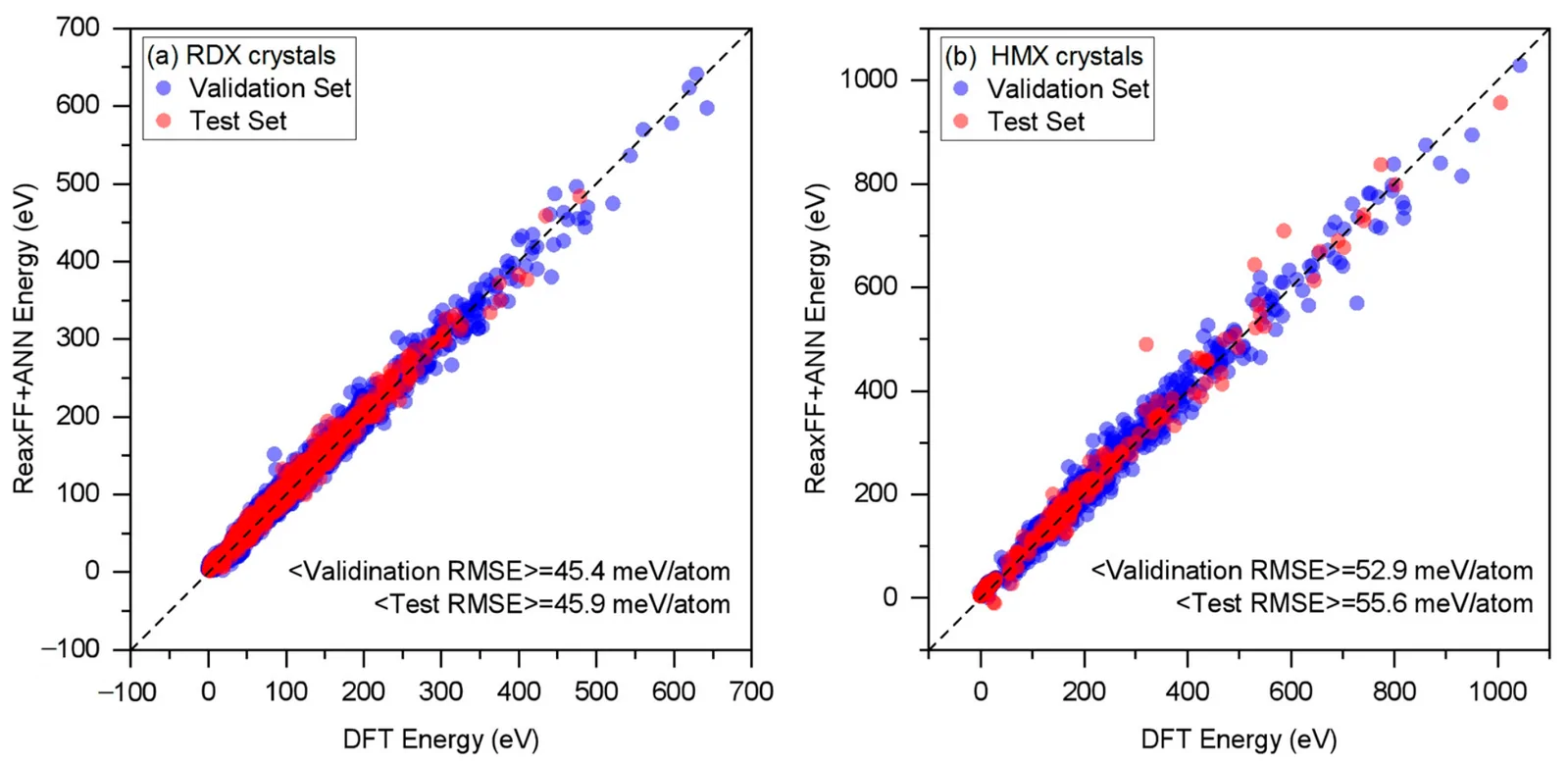
Correlation of ReaxFF + ANN energies with the corresponding DFT energies for all structures in the validation (blue dots) and test (red dots) sets based on the combined features of (**a**) RDX crystals and (**b**) HMX crystals by transfer learning.

First, Bayesian optimization was employed to search for the hyperparameters of the ANN to achieve the best model performance based on RDX crystals (Table S5 of the Supplementary Materials). Using five-fold cross-validation, we conducted ten independent model trainings on the RDX crystal database. The average errors for the validation and test sets were 45.4 meV/atom and 45.9 meV/atom, respectively (also see Table S6 of the Supplementary Materials). Figure 7a shows the comparison of the energies calculated by ReaxFF and the ANN model with DFT energies. It can be observed that the model trained with more features achieves similar performance to the model trained with a selected few features using feature screening methods, indicating that fewer features can also yield a model that meets the accuracy requirements. We selected the model with the smallest sum of errors in the test and validation sets from these trainings as the source model for transfer learning, with errors of 43.6 meV/atom and 45.4 meV/atom for the validation and test sets, respectively.

Subsequently, this model was applied to HMX crystals for fine-tuning of parameters. Similarly, 80% of the dataset was divided into the training/validation set, and the remaining 20% was used as the test set. Ten independent fine-tunings of the model parameters were performed, with five-fold cross-validation employed for each model training. During the fine-tuning process, all connection weights and biases in the neural network were adjusted using backpropagation. The average errors for the validation and test sets over the ten independent trainings were 52.9 meV/atom and 55.6 meV/atom, respectively (also see Table S6 of the Supplementary Materials). A comparison of the energies calculated by the ML model with DFT energies is shown in Figure 7b. It can be observed that the error of the transfer learning model is comparable to that of the ANN model trained solely on HMX crystals, with a slightly lower validation error. This suggests that the similar molecular configurations and stacking patterns between RDX and HMX crystals render their features transferable, and the relationships between these features are similar.

## 4. Conclusions

In summary, we have successfully developed and validated the Hierarchical “Center-Environment” (HCE) framework, marking the first feature engineering methodology specifically designed for organic molecular crystals combining structural, chemical, and physical information in a hierarchical manner. By explicitly encoding the multiscale nature of molecular packing and electronic distribution, the HCE framework addresses the long-standing challenge of descriptor incompatibility in complex molecular crystal systems.

More importantly, this study represents the first successful attempt to employ machine learning for the systematic refinement of reactive force fields (ReaxFF) via a Δ-learning strategy in predicting the cohesive energies of molecular crystals. Our results demonstrate that the synergy between HCE features and ML algorithms drastically mitigates the nonlinear errors inherent in ReaxFF, achieving near-DFT accuracy (42.2–53.7 meV/atom). The demonstrated transferability from RDX to HMX further confirms that HCE features capture fundamental physical invariants rather than merely overfitting to specific crystal structures.

Nevertheless, we acknowledge several limitations of the current implementation that point to future improvements. First, the HCE features are constructed from geometric and chemical information (bond lengths, angles, etc.) that are specific to the molecular crystal family. While the hierarchical decomposition framework is conceptually general, the specific feature set may need to be redesigned or augmented for systems with fundamentally different bonding patterns, such as covalent organic frameworks or ionic crystals. Second, the ML correction is currently trained on energy data only; force predictions (gradients) have not yet been implemented. This restricts the direct use of the corrected model in molecular dynamics simulations for on-the-fly force evaluation. Extending the HCE framework to include force training and gradient predictions is a priority direction for our future work.

Despite these limitations, this work establishes a new benchmark for force field accuracy in energetic materials and paves the way for future generalization and high-throughput screening. Given the generality of the hierarchical decomposition strategy, we anticipate that the HCE framework will serve as a foundational tool for the intelligent design of not only energetic materials but also a broad spectrum of molecular solids, including pharmaceuticals and protein design.

## Data Availability

The data supporting the findings of this study are available from the corresponding author upon reasonable request.

## Supplementary Materials

The following supporting information can be downloaded at https://www.mdpi.com/article/10.3390/molecules31162814/s1: Figure S1. 16 central features of intramolecular feature model. Figure S2. 29 environmental features of intramolecular feature model (* weight represents the weight by reciprocal of distances). Figure S3. Five central features of intermolecular feature model. Figure S4. 11 environmental features of intermolecular feature model (* weight represents the weight by reciprocal of distances). Table S1. Hyper-parameters of machine learning models of RDX crystals. Table S2. Hyper-parameters of machine learning models of HMX crystals. Table S3. MAE, RMSE (in unit of meV/atom) and R^2^ of different algorithms (ReaxFF + ML) of RDX crystals. Table S4. MAE, RMSE (in unit of meV/atom) and R^2^ of different algorithms (ReaxFF + ML) of HMX crystals. Table S5. Hyper-parameters of the ANN transfer learning model from RDX to HMX. Table S6. MAE, RMSE (in unit of meV/atom) and R^2^ of RDX crystals and HMX crystals (ReaxFF + ML) by transfer learning. Table S7. Complete regression equations, Pearson correlation coefficients (R), and RMSE (in unit of meV/atom) for all ML models on the validation and test sets.

## References

[1] Pagoria P.F., Lee G.S., Mitchell A.R., Schmidt R.D. (2002). A review of energetic materials synthesis. Thermochim. Acta.

[2] Badgujar D.M., Talawar M.B., Asthana S.N., Mahulikar P.P. (2008). Advances in science and technology of modern energetic materials: An overview. J. Hazard. Mater..

[3] Pouretedal H.R. (2026). A review on the thermal behavior of molecular explosives of RDX and HMX. J. Therm. Anal. Calorim..

[4] Zhang H., Zhang Y., Zhang X. (2026). Modeling the combustion chemistry of typical nitramine monopropellants: A critical review. FirePhysChem.

[5] Gibbs T.R., Popolato A. (1980). LASL Explosive Property Data.

[6] Chen S., Wang H., Gao Y., Huang X., Hao H. (2026). Energetic crystal engineering via intermolecular interactions: From fundamental understanding to advanced application. CrystEngComm.

[7] Price S.L. (2009). Computed crystal energy landscapes for understanding and predicting organic crystal structures and polymorphism. Acc. Chem. Res..

[8] Liu G., Gou R., Li H., Zhang C. (2018). Polymorphism of energetic materials: A comprehensive study of molecular conformers, crystal packing, and the dominance of their energetics in governing the most stable polymorph. Cryst. Growth Des..

[9] Dreger Z.A., Gupta Y.M. (2010). Phase diagram of hexahydro-1,3,5-trinitro-1,3,5-triazine crystals at high pressures and temperatures. J. Phys. Chem. A.

[10] Wang C., Zhang C., Xue X. (2022). Pressure and polymorph dependent thermal decomposition mechanism of molecular materials: A case of 1,3,5-trinitro-1,3,5-triazine. J. Phys. Chem. A.

[11] Wang C., Zhang C., You B., Xue X. (2024). Exploring the high-pressure polymorphs of flexible molecules by crystal structure prediction: A case of 1,3,5-trinitro-1,3,5-triazinane. Cryst. Growth Des..

[12] Korsunskii B.L., Aldoshin S.M., Vozchikova S.A., Golovina N.I., Chukanov N.V., Shilov G.V. (2011). A new crystalline HMX polymorph: ε-HMX. Russ. J. Phys. Chem. B.

[13] Soni P., Sarkar C., Tewari R., Sharma T.D. (2011). HMX polymorphs: Gamma to beta phase transformation. J. Energ. Mater..

[14] Myint P.C., Nichols A.L. (2016). Thermodynamics of HMX polymorphs and HMX/RDX mixtures. Ind. Eng. Chem. Res..

[15] Christopher I.L., Pulham C.R., Michalchuk A.A.L., Morrison C.A. (2023). Is the impact sensitivity of RDX polymorph dependent?. J. Chem. Phys..

[16] Goldberg I.G., Swift J.A. (2012). New insights into the metastable β form of RDX. Cryst. Growth Des..

[17] Hao L.X., Zhang B.R., Li H.N., Wang B., Dai X.G., Meng F.X., Ji C.L., Lin C.M., Wen Y.S. (2025). Physical model for thermal stress-induced phase transition of octogen in polymer-bonded explosives. Propellants Explos. Pyrotech..

[18] Gong F., Yang Z., Qian W., Liu Y., Zhang J., Ding L., Lin C., Zeng C., Yan Q.-L. (2019). Kinetics for inhibited polymorphic transition of HMX crystal after strong surface confinement. J. Phys. Chem. C.

[19] Zhou X., Li H., Zhou X., Hao S., Yan M., Zou P., Huang S., He X., Zhang C. (2022). Imparting high polymorph transition resistance to cyclotetramethylene-tetranitramine via spherulitic aggregation enabled crystal shape constraint. Chem. Eng. J..

[20] Xu J., Cheng K., Zhang H., Hao X., Xu X., Sun J., Tian Y. (2022). Effect of metal ion impurities on the crystallization and thermal properties of HMX. J. Therm. Anal. Calorim..

[21] Yang Z., Ding L., Wu P., Liu Y., Nie F., Huang F. (2015). Fabrication of RDX, HMX and CL-20 based microcapsules via in situ polymerization of melamine–formaldehyde resins with reduced sensitivity. Chem. Eng. J..

[22] Bu R., Li H., Zhang C. (2020). Polymorphic transition in traditional energetic materials: Influencing factors and effects on structure, property, and performance. Cryst. Growth Des..

[23] Wang H., Liang W.T., Wang X.Q., Mai D., Zhong C., Sun X., Dai R., Wang Z., Zheng X., Zheng W. (2024). Generation and evolution mechanism of cracks in HMX single crystals around phase transition temperature. Cryst. Growth Des..

[24] Gao C., Wang J., Zhang Y., Su H., Xu Z., Dai R., Wang Z., Zhang Z. (2021). Pressure-induced phase transition of β-RDX single crystals. J. Phys. Chem. C.

[25] Sorescu D.C., Rice B.M. (2016). RDX compression, α→γ phase transition, and shock Hugoniot calculations from density-functional-theory-based molecular dynamics simulations. J. Phys. Chem. C.

[26] Fan J., Wang P., Gao N. (2023). Pressure-dependent structure and electronic properties of energetic NTO crystals dominated by hydrogen-bonding interactions. Phys. Chem. Chem. Phys..

[27] Fan J., Wang P., Gao N. (2024). Temperature-dependent Raman spectroscopy and thermodynamic behaviors of energetic NTO crystal. Spectrochim. Acta Part A.

[28] Wang P.J., Fan J.Y., Su Y., Zhao J.J. (2020). Energetic potential of hexogen constructed by machine learning. Acta Phys. Sin..

[29] Sorescu D.C., Rice B.M., Thompson D.L. (1997). Intermolecular potential for the hexahydro-1,3,5-trinitro-1,3,5-s-triazine crystal (RDX): A crystal packing, Monte Carlo, and molecular dynamics study. J. Phys. Chem. B.

[30] Sorescu D.C., Rice B.M., Thompson D.L. (1998). A transferable intermolecular potential for nitramine crystals. J. Phys. Chem. A.

[31] Guo Y., Thompson D.L. (1999). Theoretical studies of the decomposition of RDX in liquid xenon. J. Phys. Chem. B.

[32] Liu H., Zhao J., Ji G., Gong Z., Wei D. (2006). Compressibility of liquid nitromethane in the high-pressure regime. Phys. B.

[33] Kohno Y., Ueda K., Imamura A. (1996). Molecular dynamics simulations of initial decomposition process on the unique N−N bond in nitramines in the crystalline state. J. Phys. Chem..

[34] Duan X.H., Li W.P., Pei C.H., Zhou X.Q. (2013). Molecular dynamics simulations of void defects in the energetic material HMX. J. Mol. Model..

[35] Van Duin A.C.T., Dasgupta S., Lorant F., Goddard W.A. (2001). ReaxFF: A reactive force field for hydrocarbons. J. Phys. Chem. A.

[36] Strachan A., van Duin A.C.T., Chakraborty D., Dasgupta S., Goddard W.A. (2003). Shock waves in high-energy materials: The initial chemical events in nitramine RDX. Phys. Rev. Lett..

[37] Guo F., Cheng X.L., Zhang H. (2012). Reactive molecular dynamics simulation of solid nitromethane impact on (010) surfaces induced and nonimpact thermal decomposition. J. Phys. Chem. A.

[38] Wood M.A., Van Duin A.C.T., Strachan A. (2014). Coupled thermal and electromagnetic induced decomposition in the molecular explosive α-HMX; a reactive molecular dynamics study. J. Phys. Chem. A.

[39] Liu L., Liu Y., Zybin S.V., Sun H., Goddard W.A. (2011). ReaxFF-lg: Correction of the ReaxFF reactive force field for London dispersion, with applications to the equations of state for energetic materials. J. Phys. Chem. A.

[40] Wang Q., He Q., Xiao B., Zhai D., Shen Y., Liu Y., Goddard W.A. (2024). Detailed reaction kinetics for hydrocarbon fuels: The development and application of the ReaxFFCHO-S22 force field for C/H/O systems with enhanced accuracy. J. Phys. Chem. A.

[41] Ye Z.H., Guo F., Chai C.G., Wen Y.S., Zhang Z.R., Li H.S., Cui S.X., Zhang G.Q., Wang X.C. (2024). Searching new cocrystal structures of CL-20 and HMX via evolutionary algorithm and machine learning potential. J. Mater. Inf..

[42] Behler J., Parrinello M. (2007). Generalized neural-network representation of high-dimensional potential-energy surfaces. Phys. Rev. Lett..

[43] Butler K.T., Davies D.W., Cartwright H., Isayev O., Walsh A. (2018). Machine learning for molecular and materials science. Nature.

[44] Rupp M., Tkatchenko A., Müller K.R., von Lilienfeld O.A. (2012). Fast and accurate modeling of molecular atomization energies with machine learning. Phys. Rev. Lett..

[45] Vu K., Snyder J.C., Li L., Rupp M., Chen B.F., Khelif T., Müller K.R., Burke K. (2015). Understanding kernel ridge regression: Common behaviors from simple functions to density functionals. Int. J. Quantum Chem..

[46] Bartók A.P., Payne M.C., Kondor R., Csányi G. (2010). Gaussian approximation potentials: The accuracy of quantum mechanics, without the electrons. Phys. Rev. Lett..

[47] Cao L.Z., Wang P.J., Sai L.W., Fu J., Duan X.M. (2020). Artificial neural network potential for gold clusters. Chin. Phys. B.

[48] Pei W., Wang Z., Xia W., Huang Z., Wang P., Liu Y., Zhou S., Tu Y., Zhao J. (2024). Rational design of full-color fluorescent C3N quantum dots. J. Phys. Chem. Lett..

[49] Omee S.S., Wei L., Hu J. (2024). Crystal structure prediction using neural network potential and age-fitness Pareto genetic algorithm. J. Mater. Inf..

[50] Li C.N., Liang H.P., Zhao B.Q., Wei S.H., Xie Z. (2024). Machine learning assisted crystal structure prediction made simple. J. Mater. Inf..

[51] Fang L., Qin L., Zhang L., Zhou H., He X., Ren Z., Zhang T., Liu Y. (2026). Transfer learning from homogeneous to heterogeneous: Fine-tuning a pretrained interatomic potential for multicomponent Mo alloys with localized substitutional alloying. Materials.

[52] Bartók A.P., Kondor R., Csányi G. (2013). On representing chemical environments. Phys. Rev. B.

[53] Caro M.A. (2019). Optimizing many-body atomic descriptors for enhanced computational performance of machine learning based interatomic potentials. Phys. Rev. B.

[54] Kang P.L., Shang C., Liu Z.P. (2020). Large-scale atomic simulation via machine learning potentials constructed by global potential energy surface exploration. Acc. Chem. Res..

[55] Clements R.J., Dickman J., Johal J., Martin J., Glover J., Day G.M. (2022). Roles and opportunities for machine learning in organic molecular crystal structure prediction and its applications. MRS Bull..

[56] Musil F., De S., Yang J., Campbell J.E., Day G.M., Ceriotti M. (2018). Machine learning for the structure–energy–property landscapes of molecular crystals. Chem. Sci..

[57] McDonagh D., Skylaris C.K., Day G.M. (2019). Machine-learned fragment-based energies for crystal structure prediction. J. Chem. Theory Comput..

[58] Zhao C., Chen L., Che Y., Pang Z., Wu X., Lu Y., Liu H., Day G.M., Cooper A.I. (2021). Digital navigation of energy–structure–function maps for hydrogen-bonded porous molecular crystals. Nat. Commun..

[59] Wengert S., Csányi G., Reuter K., Margraf J.T. (2021). Data-efficient machine learning for molecular crystal structure prediction. Chem. Sci..

[60] Wengert S., Csányi G., Reuter K., Margraf J.T. (2022). A hybrid machine learning approach for structure stability prediction in molecular co-crystal screenings. J. Chem. Theory Comput..

[61] Kapil V., Engel E.A. (2022). A complete description of thermodynamic stabilities of molecular crystals. Proc. Natl. Acad. Sci. USA.

[62] Xiao B., Tang Y., Liu Y. (2025). Integrating materials representations into feature engineering in machine learning for crystalline materials: From local to global chemistry-structure information coupling. WIREs Comput. Mol. Sci..

[63] Wang X., Xiao B., Li Y., Tang Y., Liu F., Chen J., Liu Y. (2020). First-principles based machine learning study of oxygen evolution reactions of perovskite oxides using a surface center-environment feature model. Appl. Surf. Sci..

[64] Li Y., Xiao B., Tang Y., Liu F., Wang X., Yan F., Liu Y. (2020). Center-environment feature model for machine learning study of spinel oxides based on first-principles computations. J. Phys. Chem. C.

[65] Li Y., Zhang X., Li T., Chen Y., Liu Y., Feng L. (2024). Accelerating materials discovery for electrocatalytic water oxidation via center-environment deep learning in spinel oxides. J. Mater. Chem. A.

[66] Li Y., Zhu R., Wang Y., Feng L., Liu Y. (2023). Center-environment deep transfer machine learning across crystal structures: From spinel oxides to perovskite oxides. npj Comput. Mater..

[67] Guo J., Xiao B., Li Y., Zhai D., Tang Y., Du W., Liu Y. (2021). Machine learning aided first-principles studies of structure stability of Co3(Al,X) doped with transition metal elements. Comput. Mater. Sci..

[68] Guo J., Xiao B., Tang Y., Li Y., Zhai D., Fan X., Liu Y. (2024). Element-configuration dependent first-principles machine learning studies of multiple alloying effects on the structure stability of Co3(Al,W). Comput. Mater. Sci..

[69] Gao J., Gao Z., Fan X., Manzhos S., Zhang T., Liu Y. (2025). Transfer learning of high-dimensional features via attention-based embedding for Ni-based superalloys. J. Appl. Phys..

[70] Xiao B., Wu Y.Q., Liu Y. (2020). Machine learning on doping of nickel-base single crystal superalloy based on first-principles calculation. Shanghai Met..

[71] Tang Y., Xiao B., Ihara M., Manzhos S., Liu Y. (2025). Effects of nonlinearity and inter-feature coupling in machine learning studies of Nb alloys with center-environment features. J. Mater. Inf..

[72] Tang Y., Xiao B., Chen J., Liu F., Du W., Guo J., Liu Y., Liu Y. (2023). Multi-component alloying effects on the stability and mechanical properties of Nb and Nb–Si alloys: A first-principles study. Metall. Mater. Trans. A.

[73] Tang Y.C., Xiao B., Chen J.H., Chen S.Z., Li Y.-H., Liu F., Du W., Shen Y.-H., Fan X., Qian Q. (2025). Machine learning with center-environment attention mechanism for multi-component Nb alloys. Trans. Nonferrous Met. Soc. China.

[74] Tang Y., Xiao B., Chen S., Qian Q., Liu Y. (2025). Predefined attention-focused mechanism using center-environment features: A machine learning study of alloying effects on the stability of Nb_5_Si_3_ alloys. Digit. Discov..

[75] Gao J., Tang Y., Fang L., Xiao B., Liu Y., Liu Y. (2025). Machine learning of energy and structure of multicomponent Nb-Si alloys via feature dimensionality reduction and augmentation. Spec. Cast. Nonferrous Alloys.

[76] Chen R., Liu F., Tang Y., Liu Y., Dong Z., Deng Z., Zhao X., Liu Y. (2022). Combined first-principles and machine learning study of the initial growth of carbon nanomaterials on metal surfaces. Appl. Surf. Sci..

[77] Akkermans R.L.C., Spenley N.A., Robertson S.H. (2013). Monte Carlo methods in Materials Studio. Mol. Simul..

[78] Day G.M., Motherwell W.D.S., Ammon H.L., Boerrigter S.X.M., Della Valle R.G., Venuti E., Dzyabchenko A., Dunitz J.D., Schweizer B., van Eijck B.P. (2005). A third blind test of crystal structure prediction. Acta Crystallogr. Sect. B.

[79] Song H.J., Li H., Zhang P., Yang Y.Q., Huang F.L. (2018). Isentropic compression loading locus of α-RDX based on its self-consistent force field. Chin. J. Energ. Mater..

[80] Thompson A.P., Aktulga H.M., Berger R., Bolintineanu D.S., Brown W.M., Crozier P.S., Veld P.J.I., Kohlmeyer A., Moore S.G., Nguyen T.D. (2022). LAMMPS—A flexible simulation tool for particle-based materials modeling at the atomic, meso, and continuum scales. Comput. Phys. Commun..

[81] Kresse G., Furthmüller J. (1996). Efficient iterative schemes for ab initio total-energy calculations using a plane-wave basis set. Phys. Rev. B.

[82] Perdew J.P., Burke K., Ernzerhof M. (1996). Generalized gradient approximation made simple. Phys. Rev. Lett..

[83] Blöchl P.E. (1994). Projector augmented-wave method. Phys. Rev. B.

[84] Fan J., Su Y., Zheng Z., Zhang Q., Zhao J. (2019). The pressure effects and vibrational properties of energetic material: Hexahydro-1,3,5-trinitro-1,3,5-triazine (α-RDX). J. Raman Spectrosc..

[85] Fan J., Su Y., Zheng Z., Zhao J. (2021). Thermal properties of energetic materials from quasi-harmonic first-principles calculations. J. Phys. Condens. Matter.

[86] Sorescu D.C., Rice B.M. (2010). Theoretical Predictions of Energetic Molecular Crystals at Ambient and Hydrostatic Compression Conditions Using Dispersion Corrections to Conventional Density Functionals (DFT-D). J. Phys. Chem. C.

