# Supplementary material for: Machine Learning-Driven Refinement of Reactive Force Fields via Hierarchical “Center-Environment” Features for Energetic Molecular Crystals
Source: Molecules. 2026 Aug 12;31(16):2814. doi: 10.3390/molecules31162814 (PMC13515906; doi:10.3390/molecules31162814)
Supplement: Supplementary file 1 [file molecules-31-02814-s001.zip › molecules-4473504-supplementary.pdf]

Supplementary Materials of

Machine Learning-Driven Refinement of Reactive Force Fields via Hierarchical "Center-Environment" Features for Energetic Molecular Crystals

Qi He [1], Pengju Wang [1,2,*], Xudong He [1], Jincang Zhang [1,2], and Yi Liu [1,2,*]

[1]Materials Genome Institute & State Key Laboratory of Materials for Advanced Nuclear Energy, Shanghai University, Shanghai 200444, China; heqi@shu.edu.cn (Q.H.); hxd_yi@shu.edu.cn (X.H.); jczhang@shu.edu.cn (J.Z.)

[2]Zhejiang Laboratory, Hangzhou 311100, China.

[*]Corresponding authors, E-mails:

wpj201222028@gmail.com (P. W.); yiliu@shu.edu.cn (Y. L.)

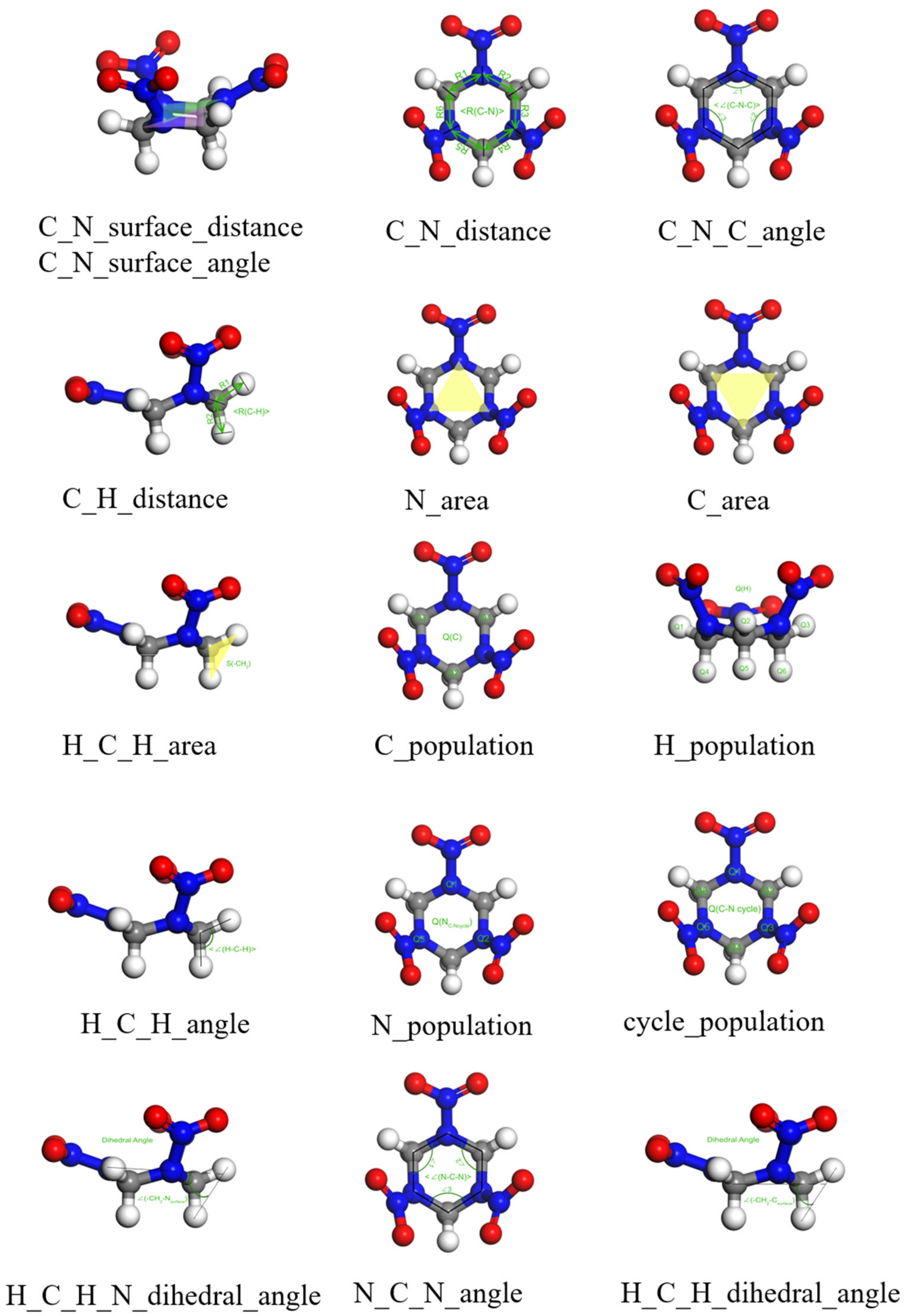

Figure S1. 16 central features of intramolecular feature model.

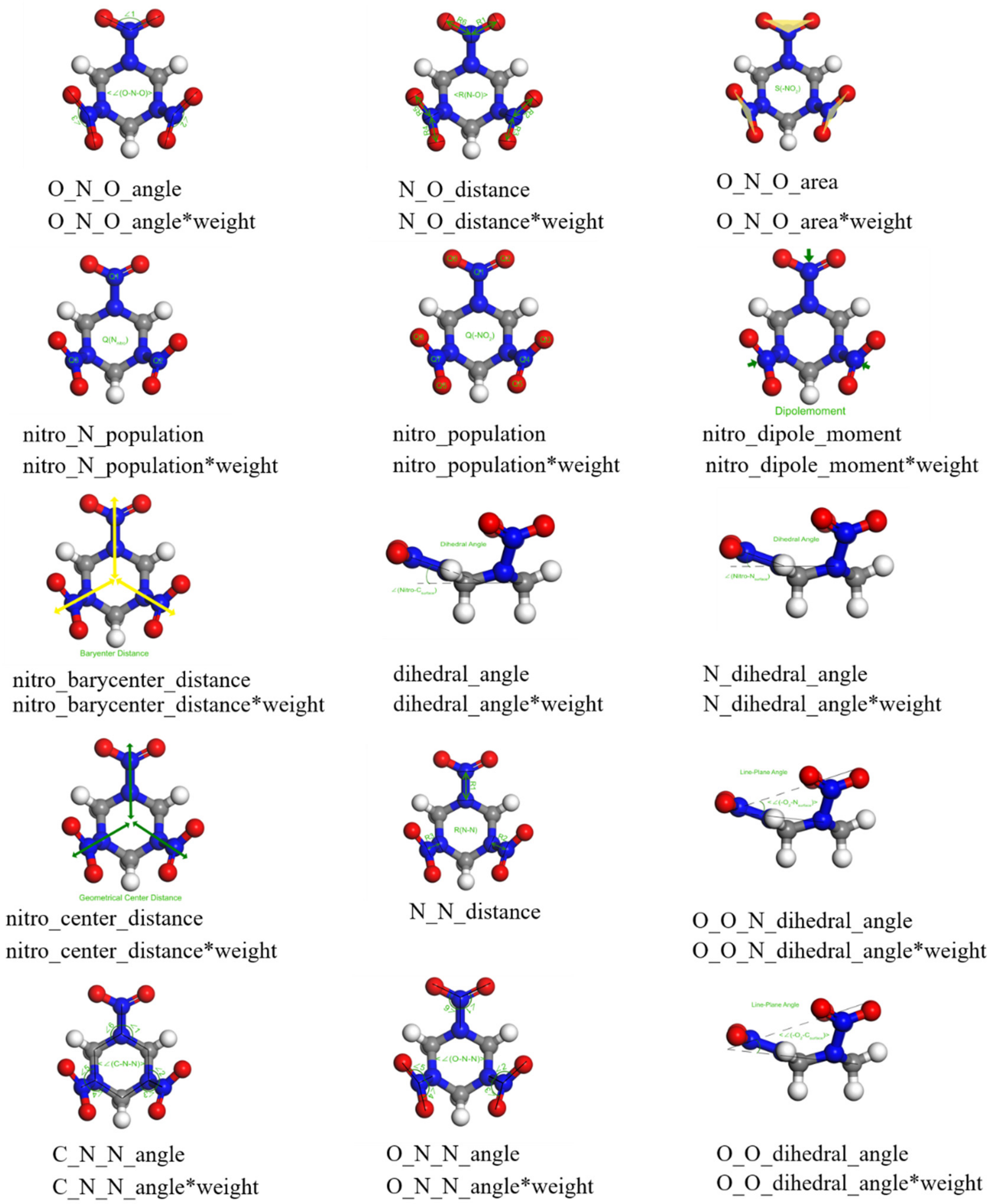

Figure S2. 29 environmental features of intramolecular feature model (*weight represents the weight by reciprocal of distances).

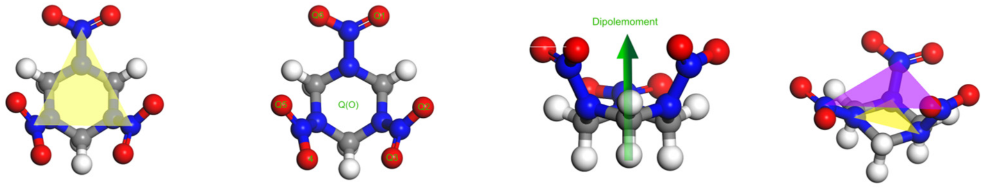

nitro_N_area O_population dipole_moment N_N_surface_angle N_N_surface_distance

Figure S3. Five central features of intermolecular feature model.

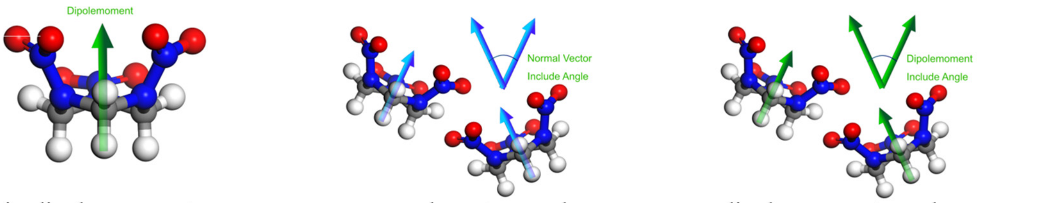

envir_dipole_moment
envir_dipole_moment*weight

normal_vector_angle
normal_vector_angle*weight

dipole_moment_angle
dipole_moment_angle*weight

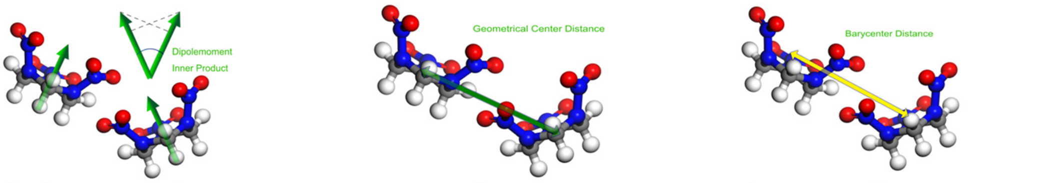

dipole_moment_dot
dipole_moment_dot*weight

center_distance

barycenter_distance
barycenter_distance*weight

Figure S4. 11 environmental features of intermolecular feature model (*weight represents the weight by reciprocal of distances).

Table S1. Hyper-parameters of machine learning models of RDX crystals.

Algorithm	Hyper-parameter	Value
ANN	neurons	50/10
	optimizer	Adam
	learning rate	1×10^{-3}
	activate function	Relu
KRR	kernel	poly
	alpha	1×10^{-4}
RF	estimators	1900
	max_depth	45
	max_features	20
SVR	kernel	poly
	C	5
	tol	1×10^{-8}
	epsilon	1×10^{-2}

Table S2. Hyper-parameters of machine learning models of HMX crystals.

Algorithm	Hyper-parameter	Value
ANN	neurons	50/10
	optimizer	Adam
	learning rate	1×10^{-3}
	activate function	Relu
KRR	kernel	linear
	alpha	1×10^{-5}
RF	estimators	1100
	max_depth	40
	max_features	35
SVR	kernel	rbf
	C	5
	tol	1×10^{-8}
	epsilon	1×10^{-2}

Table S3. MAE, RMSE (in unit of meV/atom) and R^2 of different algorithms (ReaxFF+ML) of RDX crystals.

Algorithm	Validation set			Test set		
	MAE	RMSE	R^2	MAE	RMSE	R^2
ANN	27.1	45.3 ± 1.7	0.991	26.3	45.7 ± 1.0	0.990
KRR	26.8	42.2 ± 0.6	0.992	26.9	42.4 ± 1.7	0.991
RF	26.5	43.9 ± 1.6	0.990	27.0	44.8 ± 0.3	0.991
SVR	25.7	44.9 ± 4.1	0.989	26.8	46.6 ± 1.7	0.990

Table S4. MAE, RMSE (in unit of meV/atom) and R^2 of different algorithms (ReaxFF+ML) of HMX crystals.

Algorithm	Validation set			Test set		
	MAE	RMSE	R^2	MAE	RMSE	R^2
ANN	25.8	53.6 ± 3.8	0.991	25.9	55.4 ± 5.1	0.991
KRR	31.7	60.6 ± 2.0	0.989	28.8	61.0 ± 1.0	0.992
RF	23.1	54.9 ± 2.1	0.992	23.1	57.1 ± 5.8	0.991
SVR	23.0	49.8 ± 2.8	0.993	25.8	53.7 ± 1.8	0.990

Table S5. Hyper-parameters of the ANN transfer learning model from RDX to HMX.

Algorithm	Hyper-parameter	Value
ANN	neurons	60/30
	optimizer	Adam
	learning rate	1×10^{-3}
	activate function	Relu

Table S6. MAE, RMSE (in unit of meV/atom) and R^2 of RDX crystals and HMX crystals (ReaxFF+ML) by transfer learning.

	Validation set			Test set		
	MAE	RMSE	R^2	MAE	RMSE	R^2
RDX	27.4	45.4	0.990	27.0	45.9	0.991
HMX	24.7	52.9	0.991	23.1	55.6	0.990

Table S7. Complete regression equations, Pearson correlation coefficients (R), and RMSE (in unit of meV/atom) for all ML models on the validation and test sets.

System	Model	Dataset	Regression Equation	R	RMSE
RDX	ReaxFF	All	$E_{ReaxFF} = 1.12501 \times E_{DFT} - 8.46758$	0.975	116.7
HMX	ReaxFF	All	$E_{ReaxFF} = 0.99471 \times E_{DFT} + 0.38276$	0.982	112.3
RDX	ANN	Validation	$E_{ML} = 1.00384 \times E_{DFT} - 0.43587$	0.995	45.3 ± 1.7
RDX	ANN	Test	$E_{ML} = 0.99827 \times E_{DFT} + 1.04169$	0.995	45.7 ± 1.0
RDX	KRR	Validation	$E_{ML} = 0.99423 \times E_{DFT} + 0.38036$	0.996	42.2 ± 0.6
RDX	KRR	Test	$E_{ML} = 1.01083 \times E_{DFT} - 0.29431$	0.995	42.4 ± 1.7
RDX	RF	Validation	$E_{ML} = 0.98772 \times E_{DFT} + 0.91542$	0.995	43.9 ± 1.6
RDX	RF	Test	$E_{ML} = 0.98931 \times E_{DFT} + 0.54843$	0.995	44.8 ± 0.3
RDX	SVR	Validation	$E_{ML} = 0.98519 \times E_{DFT} + 0.84222$	0.994	44.9 ± 4.1
RDX	SVR	Test	$E_{ML} = 0.99472 \times E_{DFT} + 0.21899$	0.995	46.6 ± 1.7
HMX	ANN	Validation	$E_{ML} = 0.99216 \times E_{DFT} - 0.27611$	0.995	53.6 ± 3.8
HMX	ANN	Test	$E_{ML} = 0.98597 \times E_{DFT} - 0.99635$	0.995	55.4 ± 5.1
HMX	KRR	Validation	$E_{ML} = 0.99262 \times E_{DFT} + 0.51328$	0.994	60.6 ± 2.0
HMX	KRR	Test	$E_{ML} = 1.00743 \times E_{DFT} - 0.20213$	0.996	61.0 ± 1.0
HMX	RF	Validation	$E_{ML} = 0.98290 \times E_{DFT} + 1.16573$	0.996	54.9 ± 2.1
HMX	RF	Test	$E_{ML} = 0.99200 \times E_{DFT} + 0.76548$	0.995	57.1 ± 5.8
HMX	SVR	Validation	$E_{ML} = 0.98984 \times E_{DFT} + 0.42693$	0.996	49.8 ± 2.8
HMX	SVR	Test	$E_{ML} = 0.98101 \times E_{DFT} + 0.49012$	0.995	53.7 ± 1.8
RDX	ANN_TL	Validation	$E_{ML} = 0.99030 \times E_{DFT} + 1.08144$	0.995	45.4
RDX	ANN_TL	Test	$E_{ML} = 1.00454 \times E_{DFT} + 0.08100$	0.995	45.9
HMX	ANN_TL	Validation	$E_{ML} = 0.99387 \times E_{DFT} + 1.23263$	0.995	52.9
HMX	ANN_TL	Test	$E_{ML} = 0.99948 \times E_{DFT} + 0.99118$	0.995	55.6
